# 1-(4-Bromo­benzo­yl)-2-phenyl­pyrrolidine-2-carboxamide

**DOI:** 10.1107/S1600536808003954

**Published:** 2008-02-13

**Authors:** Rafael Tamazyan, Armen Ayvazyan, Ashot Martirosyan, Gohar Harutunyan, Vahan Martirosyan

**Affiliations:** a Molecular Structure Research Center, National Academy of Sciences RA, Azatutyan Ave. 26, 375014 Yerevan, Republic of Armenia; b Institute of Fine Organic Chemistry, National Academy of Sciences RA, Azatutyan Ave. 26, 375014 Yerevan, Republic of Armenia

## Abstract

In the title compound, C_18_H_17_BrN_2_O_2_, which is a potential ­human immunodeficiency virus type 1 (HIV-1) non-nucleoside reverse transcriptase inhibitor, the pyrrolidine ring exhibits an envelope conformation. In the crystal structure, inter­molecular N—H⋯O hydrogen bonds [N⋯O = 2.861 (3) Å] link the mol­ecules into centrosymmetric dimers.

## Related literature

For related crystal structures, see: Karapetyan *et al.* (2002 ▶); Tamazyan *et al.* (2002 ▶, 2007 ▶). For details of the synthesis, see: Martirosyan *et al.* (2000 ▶, 2004 ▶). For potential pharmacological applications, see: De Clercq (1996 ▶).
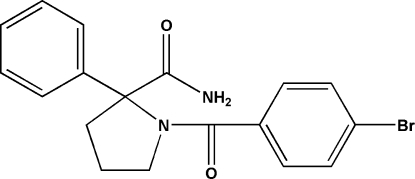


## Experimental

### 

#### Crystal data

C_18_H_17_BrN_2_O_2_
*M* *_r_* = 373.25
Monoclinic, 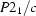

*a* = 9.5707 (19) Å
*b* = 13.738 (3) Å
*c* = 13.302 (3) Å
β = 96.99 (2)°
*V* = 1736.0 (6) Å^3^
*Z* = 4
Mo *K*α radiation
μ = 2.38 mm^−1^
*T* = 260 (2) K
0.14 mm (radius)

#### Data collection

Enraf–Nonius CAD-4 diffractometer
Absorption correction: for a sphere (*SHELXTL*; Sheldrick, 2008 ▶) *T*_min_ = 0.612, *T*_max_ = 0.617
8356 measured reflections
4180 independent reflections
2941 reflections with *I* > 2σ(*I*)
*R*_int_ = 0.050
3 standard reflections frequency: 180 min intensity decay: none

#### Refinement

*R*[*F* ^2^ > 2σ(*F* ^2^)] = 0.046
*wR*(*F* ^2^) = 0.118
*S* = 1.02
4180 reflections
216 parameters
10 restraints
H atoms treated by a mixture of independent and constrained refinement
Δρ_max_ = 0.87 e Å^−3^
Δρ_min_ = −0.89 e Å^−3^

### 

Data collection: *DATACOL* in *CAD-4* (Enraf–Nonius, 1988 ▶); cell refinement: *LS* in *CAD-4*; data reduction: *HELENA* (Spek, 1997 ▶); program(s) used to solve structure: *SHELXS97* (Sheldrick, 2008 ▶); program(s) used to refine structure: *SHELXL97* (Sheldrick, 2008 ▶); molecular graphics: *SHELXTL* (Sheldrick, 2008 ▶) and *ORTEPII* (Johnson, 1976 ▶); software used to prepare material for publication: *SHELXTL*.

## Supplementary Material

Crystal structure: contains datablocks global, I. DOI: 10.1107/S1600536808003954/cv2386sup1.cif

Structure factors: contains datablocks I. DOI: 10.1107/S1600536808003954/cv2386Isup2.hkl

Additional supplementary materials:  crystallographic information; 3D view; checkCIF report

## Figures and Tables

**Table 1:** Hydrogen-bond geometry (Å, °)

*D*—H⋯*A*	*D*—H	H⋯*A*	*D*⋯*A*	*D*—H⋯*A*
N7—H7*b*⋯O16	0.83 (2)	1.95 (3)	2.726 (3)	155 (3)
N7—H7*a*⋯O8^i^	0.86 (3)	2.00 (3)	2.861 (3)	176 (3)

Symmetry code: (i) 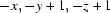.

**Fig. 1.**
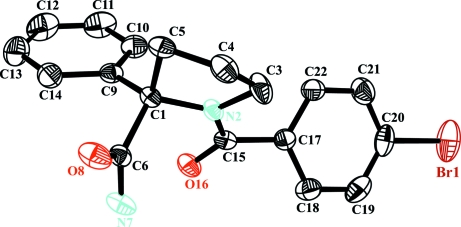
The molecular structure of (I) showing the atomic numbering and displacement ellipsoids at the 50% probability level. H atoms omitted for clarity.

**Fig. 2.**
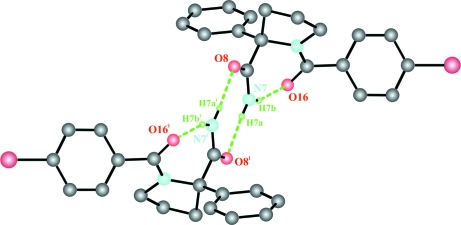
Hydrogen-bonded (dashed lines) dimer in the crystal structure of (I) [symmetry code: (i) -x, 1 - y, 1 - z]. Only H atoms participating in hydrogen-bonding are shown.

## supplementary crystallographic information

### Comment

The title compound, (I), belongs to a family of non-nucleoside reverse transcriptase inhibitors (NNRTIs), which exhibit potential HIV-1 RT inhibition properties (De Clercq, 1996).

In (I) (Fig. 1), all bond lengths and angles are in good agreement with those observed in the related compounds (Karapetyan *et al.*, 2002; Tamazyan *et al.*, 2002, 2007). Both H atoms of amide group, H7b and H7a, respectively, are involved in intra- and intermolecular N—H···O hydrogen bonds (Table 1). The latter one links the molecules into centrosymmetric dimers (Fig. 2).

### Experimental

The title compound was synthesized by cycloalkylation of N1-(3-chloropropyl)-N1-cyano(phenyl)methyl-4-bromobenzamide in phase transfer catalyses condition to 1-(4-bromobenzoyl)-2-phenyl-2-pyrrolidinecarbonitrile and then by hydrolizes with concentric sulfuric acid (Martirosyan *et al.*, 2000, 2004). The compound as synthesized is a racemic mixture of optical isomers (*R* and S) of 1-(4-bromobenzoyl)-2-phenyl-2-pyrrolidinecarboxamide molecule. The crystals were grown from methanol solution. The suitable sample with spherical shape of the size ~0.28 mm was prepared and selected for X-ray diffraction experiment.

### Refinement

All H atoms were located on a difference map. Atoms H7a and H7b were refined isotropically. C-bound H atoms were placed in idealized positions (C—H 0.93–0.97 Å) and refined as riding, with *U*_iso_ = 1.2 *U*_eq_(C).

### Crystal data

**Table:**

C_18_H_17_BrN_2_O_2_	*F*_000_ = 760
*M**_r_* = 373.25	*D*_x_ = 1.428 Mg m^−^^3^
Monoclinic, *P*2_1_/*c*	Mo *K*α radiation λ = 0.71073 Å
Hall symbol: -P 2ybc	Cell parameters from 25 reflections
*a* = 9.5707 (19) Å	θ = 13–16º
*b* = 13.738 (3) Å	µ = 2.38 mm^−^^1^
*c* = 13.302 (3) Å	*T* = 260 (2) K
β = 96.99 (2)º	Spherical, colourless
*V* = 1736.0 (6) Å^3^	0.28 × 0.28 × 0.28 × 0.14 (radius) mm
*Z* = 4	

### Data collection

**Table:**

Enraf–Nonius CAD-4 diffractometer	*R*_int_ = 0.050
Radiation source: fine-focus sealed tube	θ_max_ = 28.0º
Monochromator: graphite	θ_min_ = 2.1º
*T* = 260(2) K	*h* = −12→12
θ/2θ scans	*k* = 0→18
Absorption correction: for a sphere(SHELXTL; Sheldrick, 2008)	*l* = −17→17
*T*_min_ = 0.612, *T*_max_ = 0.617	3 standard reflections
8356 measured reflections	every 180 min
4180 independent reflections	intensity decay: none
2941 reflections with *I* > 2σ(*I*)	

### Refinement

**Table:**

Refinement on *F*^2^	Secondary atom site location: difference Fourier map
Least-squares matrix: full	Hydrogen site location: difference Fourier map
*R*[*F*^2^ > 2σ(*F*^2^)] = 0.046	H atoms treated by a mixture of independent and constrained refinement
*wR*(*F*^2^) = 0.119	*w* = 1/[σ^2^(*F*_o_^2^) + (0.0458*P*)^2^ + 1.2016*P*] where *P* = (*F*_o_^2^ + 2*F*_c_^2^)/3
*S* = 1.02	(Δ/σ)_max_ = 0.001
4180 reflections	Δρ_max_ = 0.87 e Å^−^^3^
216 parameters	Δρ_min_ = −0.89 e Å^−^^3^
10 restraints	Extinction correction: none
Primary atom site location: structure-invariant direct methods	

### Special details

**Table:**

Geometry. All e.s.d.'s (except the e.s.d. in the dihedral angle between two l.s. planes) are estimated using the full covariance matrix. The cell e.s.d.'s are taken into account individually in the estimation of e.s.d.'s in distances, angles and torsion angles; correlations between e.s.d.'s in cell parameters are only used when they are defined by crystal symmetry. An approximate (isotropic) treatment of cell e.s.d.'s is used for estimating e.s.d.'s involving l.s. planes.
Refinement. Refinement of *F*^2^ against ALL reflections. The weighted *R*-factor *wR* and goodness of fit *S* are based on *F*^2^, conventional *R*-factors *R* are based on *F*, with *F* set to zero for negative *F*^2^. The threshold expression of *F*^2^ > σ(*F*^2^) is used only for calculating *R*-factors(gt) *etc*. and is not relevant to the choice of reflections for refinement. *R*-factors based on *F*^2^ are statistically about twice as large as those based on *F*, and *R*- factors based on ALL data will be even larger.

### Fractional atomic coordinates and isotropic or equivalent isotropic displacement parameters (Å^2^)

**Table:**

	*x*	*y*	*z*	*U*_iso_*/*U*_eq_	
Br1	0.68968 (4)	1.01480 (3)	0.85408 (3)	0.07187 (17)	
C1	−0.0555 (2)	0.71666 (16)	0.64026 (16)	0.0217 (4)	
N2	0.08435 (19)	0.75423 (14)	0.68588 (13)	0.0230 (4)	
C3	0.1138 (3)	0.7265 (2)	0.79443 (18)	0.0372 (6)	
H3A	0.1112	0.7831	0.8379	0.045*	
H3B	0.2051	0.6955	0.8084	0.045*	
C4	−0.0033 (3)	0.65609 (19)	0.80987 (18)	0.0330 (5)	
H4A	−0.0257	0.6581	0.8790	0.040*	
H4B	0.0215	0.5900	0.7935	0.040*	
C5	−0.1249 (3)	0.69388 (18)	0.73599 (17)	0.0277 (5)	
H5A	−0.1656	0.7520	0.7620	0.033*	
H5B	−0.1977	0.6449	0.7224	0.033*	
C6	−0.0375 (2)	0.62094 (16)	0.58089 (16)	0.0225 (5)	
N7	0.0475 (3)	0.62518 (17)	0.50977 (16)	0.0310 (5)	
H7A	0.060 (3)	0.572 (2)	0.477 (2)	0.026 (7)*	
H7B	0.089 (3)	0.677 (2)	0.502 (2)	0.038 (8)*	
O8	−0.1011 (2)	0.54709 (12)	0.60012 (14)	0.0357 (4)	
C9	−0.1430 (2)	0.78995 (16)	0.57304 (17)	0.0245 (5)	
C10	−0.1242 (3)	0.88979 (18)	0.5831 (2)	0.0333 (5)	
H10	−0.0515	0.9139	0.6291	0.040*	
C11	−0.2120 (3)	0.9539 (2)	0.5257 (2)	0.0448 (7)	
H11	−0.1975	1.0206	0.5328	0.054*	
C12	−0.3217 (3)	0.9190 (2)	0.4578 (2)	0.0484 (8)	
H12	−0.3811	0.9621	0.4193	0.058*	
C13	−0.3422 (3)	0.8208 (2)	0.4475 (2)	0.0454 (7)	
H13	−0.4160	0.7973	0.4021	0.055*	
C14	−0.2534 (3)	0.75578 (19)	0.50447 (19)	0.0341 (6)	
H14	−0.2680	0.6891	0.4966	0.041*	
C15	0.1780 (2)	0.79997 (16)	0.63414 (17)	0.0244 (5)	
O16	0.16487 (18)	0.80521 (12)	0.54045 (12)	0.0298 (4)	
C17	0.3019 (2)	0.84793 (17)	0.69371 (18)	0.0264 (5)	
C18	0.4350 (3)	0.8311 (2)	0.6659 (2)	0.0356 (6)	
H18	0.4463	0.7867	0.6148	0.043*	
C19	0.5505 (3)	0.8797 (2)	0.7137 (2)	0.0441 (7)	
H19	0.6397	0.8679	0.6955	0.053*	
C20	0.5317 (3)	0.9461 (2)	0.7890 (2)	0.0412 (7)	
C21	0.4003 (3)	0.9652 (2)	0.8166 (2)	0.0410 (6)	
H21	0.3892	1.0110	0.8666	0.049*	
C22	0.2853 (3)	0.91537 (19)	0.7691 (2)	0.0337 (6)	
H22	0.1962	0.9271	0.7877	0.040*	

### Atomic displacement parameters (Å^2^)

**Table:**

	*U*^11^	*U*^22^	*U*^33^	*U*^12^	*U*^13^	*U*^23^
Br1	0.0470 (2)	0.0818 (3)	0.0793 (3)	−0.02922 (18)	−0.02262 (18)	0.0072 (2)
C1	0.0249 (10)	0.0220 (11)	0.0184 (10)	−0.0011 (8)	0.0038 (8)	−0.0017 (8)
N2	0.0263 (9)	0.0274 (10)	0.0154 (9)	−0.0030 (8)	0.0033 (7)	−0.0008 (7)
C3	0.0455 (15)	0.0498 (16)	0.0152 (11)	−0.0080 (12)	−0.0002 (10)	0.0018 (11)
C4	0.0441 (14)	0.0370 (14)	0.0188 (11)	−0.0031 (11)	0.0070 (10)	0.0039 (10)
C5	0.0329 (12)	0.0292 (12)	0.0229 (11)	0.0000 (10)	0.0108 (9)	−0.0012 (9)
C6	0.0273 (11)	0.0230 (11)	0.0170 (10)	0.0020 (9)	0.0014 (8)	0.0005 (8)
N7	0.0464 (13)	0.0213 (11)	0.0280 (11)	−0.0039 (10)	0.0159 (9)	−0.0048 (9)
O8	0.0492 (11)	0.0245 (9)	0.0365 (10)	−0.0080 (8)	0.0176 (8)	−0.0047 (7)
C9	0.0266 (11)	0.0263 (11)	0.0216 (11)	0.0033 (9)	0.0070 (9)	0.0019 (9)
C10	0.0359 (13)	0.0281 (13)	0.0367 (14)	0.0033 (10)	0.0076 (11)	0.0004 (11)
C11	0.0519 (17)	0.0289 (13)	0.0556 (19)	0.0102 (12)	0.0146 (15)	0.0123 (13)
C12	0.0484 (17)	0.0498 (18)	0.0472 (17)	0.0196 (14)	0.0065 (14)	0.0200 (14)
C13	0.0398 (15)	0.0563 (19)	0.0375 (16)	0.0111 (13)	−0.0064 (12)	0.0058 (13)
C14	0.0359 (13)	0.0332 (13)	0.0324 (13)	0.0040 (11)	0.0011 (11)	−0.0017 (11)
C15	0.0282 (11)	0.0221 (11)	0.0237 (11)	0.0022 (9)	0.0063 (9)	−0.0015 (9)
O16	0.0383 (9)	0.0314 (9)	0.0208 (8)	−0.0058 (7)	0.0078 (7)	−0.0010 (7)
C17	0.0257 (11)	0.0270 (11)	0.0267 (11)	0.0000 (9)	0.0042 (9)	0.0001 (10)
C18	0.0310 (13)	0.0372 (14)	0.0398 (15)	0.0024 (11)	0.0095 (11)	−0.0016 (11)
C19	0.0244 (12)	0.0528 (17)	0.0552 (18)	0.0022 (11)	0.0060 (12)	0.0068 (15)
C20	0.0299 (13)	0.0445 (15)	0.0453 (16)	−0.0118 (11)	−0.0107 (11)	0.0070 (13)
C21	0.0430 (15)	0.0394 (15)	0.0392 (15)	−0.0072 (12)	−0.0001 (12)	−0.0086 (12)
C22	0.0287 (12)	0.0357 (13)	0.0374 (14)	−0.0013 (10)	0.0064 (10)	−0.0093 (11)

### Geometric parameters (Å, °)

**Table:**

Br1—C20	1.899 (3)	C10—C11	1.382 (4)
C1—N2	1.493 (3)	C10—H10	0.9300
C1—C9	1.527 (3)	C11—C12	1.385 (5)
C1—C5	1.538 (3)	C11—H11	0.9300
C1—C6	1.554 (3)	C12—C13	1.368 (5)
N2—C15	1.350 (3)	C12—H12	0.9300
N2—C3	1.487 (3)	C13—C14	1.392 (4)
C3—C4	1.513 (4)	C13—H13	0.9300
C3—H3A	0.9700	C14—H14	0.9300
C3—H3B	0.9700	C15—O16	1.239 (3)
C4—C5	1.520 (3)	C15—C17	1.496 (3)
C4—H4A	0.9700	C17—C18	1.388 (3)
C4—H4B	0.9700	C17—C22	1.389 (3)
C5—H5A	0.9700	C18—C19	1.378 (4)
C5—H5B	0.9700	C18—H18	0.9300
C6—O8	1.226 (3)	C19—C20	1.383 (4)
C6—N7	1.321 (3)	C19—H19	0.9300
N7—H7A	0.86 (3)	C20—C21	1.378 (4)
N7—H7B	0.83 (3)	C21—C22	1.383 (4)
C9—C10	1.388 (3)	C21—H21	0.9300
C9—C14	1.391 (3)	C22—H22	0.9300
			
N2—C1—C9	114.23 (18)	C11—C10—C9	121.0 (3)
N2—C1—C5	100.93 (17)	C11—C10—H10	119.5
C9—C1—C5	110.99 (18)	C9—C10—H10	119.5
N2—C1—C6	110.43 (17)	C10—C11—C12	120.1 (3)
C9—C1—C6	110.32 (17)	C10—C11—H11	120.0
C5—C1—C6	109.54 (18)	C12—C11—H11	120.0
C15—N2—C3	123.7 (2)	C13—C12—C11	119.6 (3)
C15—N2—C1	124.89 (18)	C13—C12—H12	120.2
C3—N2—C1	111.14 (18)	C11—C12—H12	120.2
N2—C3—C4	103.87 (19)	C12—C13—C14	120.6 (3)
N2—C3—H3A	111.0	C12—C13—H13	119.7
C4—C3—H3A	111.0	C14—C13—H13	119.7
N2—C3—H3B	111.0	C9—C14—C13	120.3 (3)
C4—C3—H3B	111.0	C9—C14—H14	119.8
H3A—C3—H3B	109.0	C13—C14—H14	119.8
C3—C4—C5	102.4 (2)	O16—C15—N2	123.2 (2)
C3—C4—H4A	111.3	O16—C15—C17	118.9 (2)
C5—C4—H4A	111.3	N2—C15—C17	117.9 (2)
C3—C4—H4B	111.3	C18—C17—C22	119.5 (2)
C5—C4—H4B	111.3	C18—C17—C15	118.6 (2)
H4A—C4—H4B	109.2	C22—C17—C15	121.5 (2)
C4—C5—C1	103.39 (19)	C19—C18—C17	120.5 (3)
C4—C5—H5A	111.1	C19—C18—H18	119.8
C1—C5—H5A	111.1	C17—C18—H18	119.8
C4—C5—H5B	111.1	C18—C19—C20	119.1 (3)
C1—C5—H5B	111.1	C18—C19—H19	120.5
H5A—C5—H5B	109.1	C20—C19—H19	120.5
O8—C6—N7	123.4 (2)	C21—C20—C19	121.5 (2)
O8—C6—C1	120.3 (2)	C21—C20—Br1	119.0 (2)
N7—C6—C1	116.3 (2)	C19—C20—Br1	119.5 (2)
C6—N7—H7A	117.1 (18)	C20—C21—C22	119.0 (3)
C6—N7—H7B	119 (2)	C20—C21—H21	120.5
H7A—N7—H7B	124 (3)	C22—C21—H21	120.5
C10—C9—C14	118.4 (2)	C21—C22—C17	120.4 (2)
C10—C9—C1	122.7 (2)	C21—C22—H22	119.8
C14—C9—C1	118.7 (2)	C17—C22—H22	119.8
			
C9—C1—N2—C15	−49.1 (3)	C1—C9—C10—C11	−175.3 (2)
C5—C1—N2—C15	−168.3 (2)	C9—C10—C11—C12	0.7 (4)
C6—C1—N2—C15	75.9 (3)	C10—C11—C12—C13	−0.2 (5)
C9—C1—N2—C3	136.3 (2)	C11—C12—C13—C14	−0.3 (5)
C5—C1—N2—C3	17.2 (2)	C10—C9—C14—C13	0.1 (4)
C6—C1—N2—C3	−98.6 (2)	C1—C9—C14—C13	175.0 (2)
C15—N2—C3—C4	−165.7 (2)	C12—C13—C14—C9	0.3 (4)
C1—N2—C3—C4	9.0 (3)	C3—N2—C15—O16	164.8 (2)
N2—C3—C4—C5	−31.7 (3)	C1—N2—C15—O16	−9.1 (3)
C3—C4—C5—C1	43.1 (2)	C3—N2—C15—C17	−16.3 (3)
N2—C1—C5—C4	−36.6 (2)	C1—N2—C15—C17	169.74 (19)
C9—C1—C5—C4	−158.09 (19)	O16—C15—C17—C18	−48.8 (3)
C6—C1—C5—C4	79.8 (2)	N2—C15—C17—C18	132.3 (2)
N2—C1—C6—O8	125.4 (2)	O16—C15—C17—C22	125.0 (3)
C9—C1—C6—O8	−107.4 (2)	N2—C15—C17—C22	−53.9 (3)
C5—C1—C6—O8	15.1 (3)	C22—C17—C18—C19	1.0 (4)
N2—C1—C6—N7	−55.3 (3)	C15—C17—C18—C19	174.9 (2)
C9—C1—C6—N7	71.9 (3)	C17—C18—C19—C20	−0.6 (4)
C5—C1—C6—N7	−165.6 (2)	C18—C19—C20—C21	−0.5 (4)
N2—C1—C9—C10	−23.9 (3)	C18—C19—C20—Br1	−179.2 (2)
C5—C1—C9—C10	89.4 (3)	C19—C20—C21—C22	1.2 (4)
C6—C1—C9—C10	−149.0 (2)	Br1—C20—C21—C22	179.9 (2)
N2—C1—C9—C14	161.4 (2)	C20—C21—C22—C17	−0.8 (4)
C5—C1—C9—C14	−85.3 (3)	C18—C17—C22—C21	−0.3 (4)
C6—C1—C9—C14	36.3 (3)	C15—C17—C22—C21	−174.0 (2)
C14—C9—C10—C11	−0.6 (4)		

### Hydrogen-bond geometry (Å, °)

**Table:**

*D*—H···*A*	*D*—H	H···*A*	*D*···*A*	*D*—H···*A*
N7—H7b···O16	0.83 (2)	1.95 (3)	2.726 (3)	155 (3)
N7—H7a···O8^i^	0.86 (3)	2.00 (3)	2.861 (3)	176 (3)

Symmetry codes:  (i) −*x*, −*y*+1, −*z*+1.
